# Stage and treatment variation with age in postmenopausal women with breast cancer: compliance with guidelines

**DOI:** 10.1038/sj.bjc.6601742

**Published:** 2004-03-30

**Authors:** L Wyld, D K Garg, I D Kumar, H Brown, M W R Reed

**Affiliations:** 1 Academic Surgical Oncology Unit, University of Sheffield, K Floor, Royal Hallamshire Hospital, Glossop Road, Sheffield S10 2JF, UK

**Keywords:** elderly, breast cancer, stage, treatment

## Abstract

Breast cancer-specific mortality is static in older women despite having fallen in younger age groups, possibly due to lack of screening and differences in treatment. This study compared stage and treatment between two cohorts of postmenopausal women (55–69 *vs* >70 years) in a single cancer network over 6 months. A total of 378 patients were studied (>70: *N*=167, 55–69 years: *N*=210). Older women presented with more advanced disease (>70: metastatic/locally advanced 12%, 55–69 years: 3%, *P*<0.01). Those with operable cancer had a worse prognosis (Nottingham Prognostic Index (NPI) >70: median NPI 4.4, 55–69 years: 4.25, *P*<0.03). These stage differences were partially explained by higher screening rates in the younger cohort. Primary endocrine therapy was used in 42% of older patients compared with 3% in the younger group (*P*<0.001). Older women with cancers suitable for breast conservation were more likely to choose mastectomy (>70: 57.5% mastectomy rate *vs* 55–69 years: 20.6%, *P*<0.01). Nodal surgery was less frequent in older patients (>70: 6.7% no nodal surgery, 55–69 years: 0.5%, *P*<0.01) and was more likely to be inadequate (>70: 10.7% <4 nodes excised, 55–69 years: 3.4%, *P*<0.02). In summary, older women presented with more advanced breast cancer, than younger postmenopausal women and were treated less comprehensively.

Breast cancer incidence increases with age and, as the UK population ages, is becoming more common (Quinn *et al*, 2001). Although the overall mortality rate for breast cancer is declining, on an age-specific basis, the mortality rate in the elderly has been static (Quinn *et al*, 2001). This may reflect a number of factors such as the lack of screening in older woman resulting in a more advanced stage at presentation (Tabar *et al*, 2003), or differences in treatment with age, which have previously been demonstrated in both the UK and USA (Golledge *et al*, 2000; Yancik *et al*, 2001).

## STAGE DIFFERENCES AT PRESENTATION

Breast screening in the UK is presently available to all women between 50 and 65 years by automatic recall and on demand for women over this age. However, uptake rates for voluntary breast screening in older women are low (with only 10% of all screen detected cancer arising in the over 65 years age group in the UK (NHS Breast Screening Program Audit, 2003). Screening reduces the stage at presentation of breast cancer in the under 70 years age group and improves breast cancer survival (Tabar *et al*, 2003). In the over-70 years age group the evidence that screening reduces mortality is limited, but there may be some benefit (Van Dijck *et al*, 1997). The benefit may be less in older women because although the incidence of breast cancer is greater, breast cancer-specific mortality is lower due to increased nonbreast cancer deaths. For example, 73% of deaths in breast cancer patients in the 50–54 year age group are due to breast cancer compared to only 29% of deaths in women over 85 years (Diab *et al*, 2000). This dilutes any survival benefit from breast cancer treatment so differences may be difficult to detect. Stage at presentation also differs in older women because they are less likely to self-examine (Siahpush and Singh, 2002) and therefore the size of the primary cancer is greater at diagnosis (Diab *et al*, 2000; Golledge *et al*, 2000), and the incidence of advanced disease may be greater (Yancik *et al*, 1989).

## TREATMENT DIFFERENCES

Ideally, the treatment for breast cancer should be evidence based and tailored to the patient's wishes and physical limitations. Older patients have a greater incidence of comorbidity than younger patients (Yancik *et al*, 2001) and therefore treatment options may differ.

### Primary endocrine therapy

There is greater utilisation of primary endocrine therapy, PET (i.e. tamoxifen as sole treatment) in older women. However, the five randomised controlled trials which have compared tamoxifen *vs* surgery have demonstrated better local control with surgery than with tamoxifen (Robertson *et al*, 1992; Gazet *et al*, 1994; Bates *et al*, 2001; Fentiman *et al*, 2003; Mustacchi *et al*, 2003). However, only one of these studies showed a reduction in survival with tamoxifen after 12 years of follow-up (Bates *et al*, 2001). Therefore, studies indicate that primary endocrine therapy is only justified in patients with ER-positive tumours and significant comorbidity preventing surgical treatment. Despite this evidence, use of primary endocrine therapy is still widespread in the UK (Monypenny, 2003) with almost 20% of patients aged 65–79 years receiving PET and almost 60% of women over 80 years. However it should be noted that the patients recruited into the above randomised trials were all fit for surgery. In a population study, there will be some women with severe comorbidity, where surgery and anaesthesia may be associated with significant risks of morbidity and mortality and life expectancy would be limited. In such patients, PET may be the appropriate treatment.

### Adjuvant chemotherapy

There is limited evidence for the role of adjuvant chemotherapy in the treatment of older women with breast cancer. The Early Breast Cancer Trialist's Collaborative Group indicated a similar magnitude of response to adjuvant chemotherapy in patients over 70 years compared to those between 60 and 69 years, but this did not reach significance, probably due to low numbers in this age group (EBCTCG, 1998). More recent evidence suggests that chemotherapy for oestrogen receptor-negative cancers in the elderly may confer a survival advantage (International Breast Cancer Study Group, 2002). At present, most UK Units do not routinely prescribe adjuvant chemotherapy for breast cancer in older women due to this reduced evidence of benefit and increased toxicity.

### Adjuvant radiotherapy

Radiotherapy after breast-conservation surgery reduces the rate of local recurrence from 35 to 10–12% and may reduce mortality slightly (EBCTCG, 2000). There is evidence that local recurrence rates may be lower in older women (Benhaim *et al*, 2000) and a randomised trial is evaluating the role of adjuvant radiotherapy in older patients with good prognosis breast cancer (PRIME Trial, Kunkler *et al*, 2001). Despite the lack of evidence from randomised controlled trials there may be a tendency to omit radiotherapy after breast-conservation surgery in older patients (Reed and Morrison, 1989).

There are little data about the use of chest wall radiotherapy after mastectomy in older women, although in younger women local recurrence rates are reduced by two-thirds. National Guidelines advise its use if there is a high risk of local recurrence (NICE, 2002), such as if there is margin involvement, heavy nodal disease (>4 positive nodes) or a large (T3) tumour.

### Type of surgery to the breast

Studies demonstrate wide variation in the proportion of mastectomies performed in older patients. Some studies indicate that older women are less likely to be treated by mastectomy than by breast conservation (Diab *et al*, 2000; Gajdos *et al*, 2001) others that they are more likely (Monypenny, 2003) and some that there is no difference (Grube *et al*, 2001). Certainly, the most recent UK data favour mastectomy as the most common choice for older women (Monypenny, 2003). This may be because older women present with larger primary cancers (Diab *et al*, 2000; Golledge *et al*, 2000), making breast-conservation inappropriate, or may be less concerned with body image and choose the more simple therapeutic option of mastectomy.

### Axillary surgery

There is a tendency to perform less-extensive axillary surgery in older women (Feigelson *et al*, 1996; Brenin *et al*, 1999). Sampling or sentinel node biopsy may provide an alternative to axillary clearance but a positive sample may necessitate further surgery or radiotherapy with the potential for greater morbidity. Furthermore, the omission of axillary surgery means that the 35% of patients who have nodal disease may develop regional disease progression.

There are therefore many ways in which the treatment of older patients with breast cancer may differ from that of younger patients, some justifiable, others less so.

The aim of the current study was to examine both the stage at diagnosis and primary and adjuvant treatments of two cohorts of postmenopausal women from a single cancer network in the UK over a 6-month period. Women over the age of 70 years were compared with women of 55–69 years. National and local protocol adherence was assessed for both age groups and reasons for any variance were examined.

A further aim of this study was to evaluate the proportion of older patients who would be eligible for recruitment to a trial of adjuvant chemotherapy based on prognosis and comorbidity.

## METHODS

All patients presenting with invasive or noninvasive breast cancer over a 6-month period in a single UK Regional Cancer Network (North Trent) were included in this study. The region comprises the Sheffield Cancer Centre and five associated Cancer Units. Cases were identified from multidisciplinary team records, which prospectively record 99% of all breast cancers in the region. Patients included in the study were aged 55 years or over at the time of diagnosis. Case notes were examined to obtain data concerning comorbid conditions, drug history, clinical features of the primary cancer, type of primary and adjuvant treatment and stage and histological features of the tumour. The Nottingham Prognostic Index was recorded for patients who underwent surgery to both breast and axilla (NPI, a prognostic scoring system based on the size of primary tumour, grade and nodal status, Haybittle *et al*, 1982).

The oestrogen receptor status of the tumour was recorded where available using the McCarty H score (score between 0 and 300 reflecting the percentage of cells staining for oestrogen receptors and the intensity of the staining, McCarty *et al*, 1983). Treatment related side effects were also recorded.

The Region has guidelines for the management of breast cancer (based on UK National Guidelines, National Institute for Clinical Excellence, BASO, 1998; and NICE, 2002) and all treatment choices were compared with these guidelines to assess compliance with the guidelines or whether valid reasons for noncompliance were documented.

Statistical analysis was by χ^2^ or Mann–Whitney U test. Statistical significance was accepted at *P*<0.05.

All data are represented as percentages or median plus range.

## RESULTS

A total of 378 patient records were examined between March 2002 and August 2002. Of these, 210 were in the age range 55–69 years, with a median age of 62 years (range 55–69 years) and 167 were in the age range 70 years and over, with a median age of 78.9 years (range 70–98 years). Overall, there were 280 patients with symptomatic breast cancer and 98 referred from breast screening. Very few of the over 70 years age group were screen detected, with seven out of 167 (4.1%) in the older group compared with 91 of 210 (43.3%) in the younger patients.

### Diagnostic process

National Guidelines recommend the use of triple assessment for the diagnosis of breast cancer (combination of clinical examination, mammography (± ultrasound) and biopsy (BASO, 1998; NICE, 2002). Older women were less likely to have full triple assessment and were less likely to undergo either mammography or biopsy (core/FNA/surgical), (*P*<0.05, Table 1

**Table 1:** Summary of percentage compliance with guidelines by age group

**Guideline**	**Guideline source**	**% compliance 55–69 years**	**% compliance >70 years**	**Statistical significance**
Triple assessment complete	1,2,3	98.1	81.4	*P*<0.001
Mammogram performed	1,2,3	98.6	85.6	*P*<0.001
Biopsy performed, (FNA, core or surgical)	1,2,3	100	97	*P*<0.02
Surgical treatment should not be withheld on grounds of age alone	1,2	100	97.6	*P*<0.05
Axillary staging with minimum of four nodes sampled	1,2,3	96.1	83.1	*P*<0.001
Radiotherapy after breast-conservation therapy	1,2,3	94.7	80	*P*<0.05
Radiotherapy after mastectomy if indicated^a^	3	97	70	*P*<0.01
Adjuvant tamoxifen (ER+ cancer)	1,2,3	97.8	98	NS

Statistical significance tested with χ^2^. NS=no significant difference.

a Local Protocol Indications for chest wall radiotherapy after mastectomy; Tumour >5 cm diameter and/or close/involved deep margin and/or nodal disease. 1=NICE, 2002, 2=BASO, 1998, 3=Local guidelines.

).

### Stage at presentation

Full histopathological staging was available in 111 (66.5%) of the older age group compared with 199 (94.8%) of the younger age group (*P*<0.001). This was because a larger proportion of older women either did not receive surgery (treatment with PET) or had omission of axillary surgery.

### Advanced cancer

Older women were more likely to present with advanced disease (both locally advanced, inoperable primary disease or metastatic disease, *P*<0.01, Table 2

**Table 2:** Stage at presentation of breast cancer

**Stage at presentation**	**All patients (%)**		**Symptomatic patients (%)**	
Age group (years)	>70	55–69	>70	55-69
Total number of patients	167 (100)	210 (100)	160 (100)	119 (100)
DCIS	3 (1.8)^a^	14 (6.6)	3 (1.9)	3 (2.5)
Primary operable breast cancer (stages I and II)	144 (86.2)	189 (90)	144 (90)	112 (94.1)
Locally advanced, Stage III	9 (5.4)^a^	3 (1.4)	9 (5.6)	2 (1.7)
Metastatic, Stage IV	11 (6.6)^a^	4 (1.9)	11 (6.9)^a^	2 (1.7)

It was not possible to discriminate stage I and II adequately for this study due to the high percentage of patients in the older age group who received no surgery or inadequate axillary surgery.

a Denotes statistically significant difference from value in younger age group, *P*<0.05.

). This difference persisted when screen detected cancers were excluded and symptomatic patients only were analysed for advanced disease (inoperable and metastatic).

### Primary operable breast cancer

**Figure 1:**
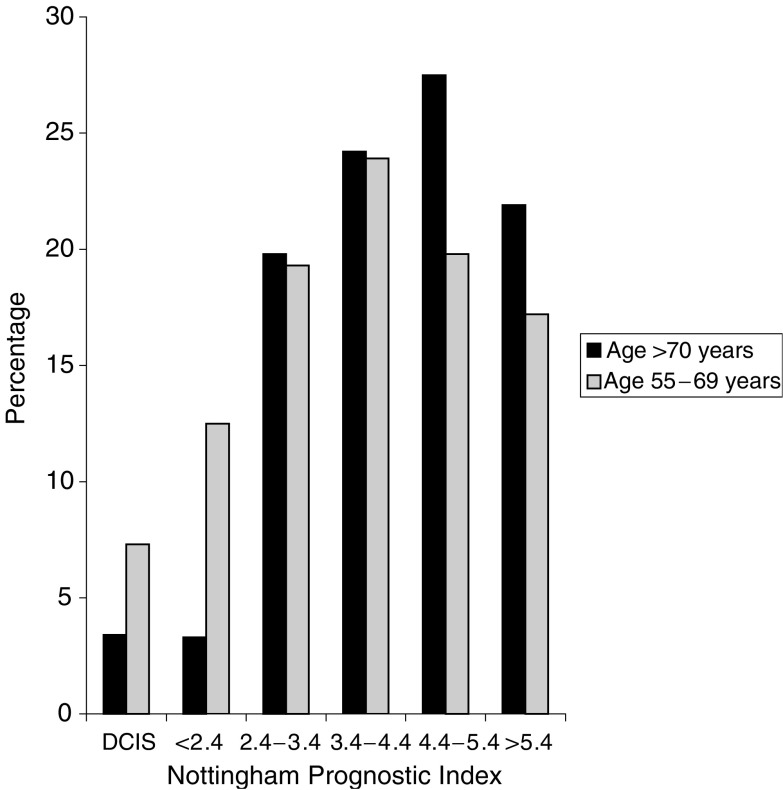
Prognostic group according to age in patients treated surgically for breast cancer. Graph shows percentage of patients in both age groups who were treated with surgery, including some form of axillary surgery for those with invasive carcinoma. These data exclude those in whom axillary staging was omitted or inadequate. DCIS: ductal carcinoma *in situ*. Nottingham Prognostic Index is the sum of the grade (1–3), the nodal status (no nodal disease=1, up to three nodes involved=2, four or more nodes involved=3) and the size of the tumour in cm divided by 5). The higher the score, the worse the prognosis.

There was a significant stage difference in the percentage presenting with primary operable cancer with good, excellent or moderate prognosis tumours favouring the younger age group and poor prognosis tumours more frequent in the older age group (Figure 1

). The median Nottingham Prognostic Index (NPI) for the older age group was 4.4 *vs* 4.25 in the 55–69 age group (*P*<0.03). This difference in NPI disappeared when screen detected cancers were excluded. Breakdown of NPI into its components (grade, tumour size, nodal disease), revealed that the difference in NPI was due to older patients having larger primary cancers (both on clinical and histopathological evaluation). Older patients also had higher-grade tumours and more nodal disease, but these differences were not significant (Table 3

**Table 3:** Characteristics of primary cancer according to age group

	**Age 55–69 years**		**Age >70 years**	
	**No. evaluable**	**Median + range**	**No. evaluable**	**Median + range**
Size of primary cancer (clinical assessment)	151	18 mm (0–200 mm)	129	26 mm^a^ (0–90 mm)
Size of primary cancer (histological assessment	96	17.3 mm (1–134 mm)	102	23 mm^a^ (4–100 mm)
				
	**No. evaluable**	**Number of patients (%)**	**No. evaluable**	**Number of patients (%)**
Grade 1	180	33 (18.3)	89	14 (15.7) ns
Grade 2	180	75 (41.7)	89	33 (37.1) ns
Grade 3	180	69 (38.3)	89	41 (46.1) ns
Nodes negative	181	124 (68.5)	82	50 (61.7) ns
Nodes positive	181	57 (31.5)	82	31 (38.3) ns

a Denotes statistically significant difference between older and younger age groups, *P*<0.002. ns: no significant difference.

).

### Ductal carcinoma *in situ* (DCIS)

There was a significantly greater incidence of DCIS in the younger age group, with 14 women in the 55–69 age group (7.3%) compared to three in the over 70 age group (3.4%, *P*<0.05, Table 2). Again this difference disappeared when screen-detected cases were excluded.

### Oestrogen receptor status

Women in the older age group had tumours with significantly greater oestrogen receptor staining (*P*<0.007). The median H score in the older age group was 204 (range 0–270), compared to 180 (range 0–276) in the 55–69 year age group.

### Treatment

#### Treatment variance between hospitals

Comparison of treatment practices between units was performed and showed no significant differences with any of the Units, although patient numbers were small in some subgroups (data not shown).

#### Primary endocrine therapy

There are no guidelines relating to the use of PET as sole treatment for breast cancer (BASO, 1998; or NICE, 2002). Local control is significantly worse, but there is little evidence of mortality difference. Most authors advise its use only for women with ER-positive tumours who are unfit for surgery (Fentiman *et al*, 2003). Significantly more patients were treated with primary endocrine therapy (70 out of 167, 41.9%), in the over 70 age group compared to the younger age group (six out of 210, 2.8%, *P*<0.00). The majority of patients had a clear indication for PET in both age groups with most having either metastatic or inoperable disease or significant comorbidity likely to reduce life expectancy (Table 4

**Table 4:** Primary endocrine therapy

**Treatment variable**	**55–69 years**	**>70 years**
Total number of patients	210	167
Number treated with Primary Endocrine Therapy	6 (2.8%)	70 (40.3%)
Metastatic or LAPC	3	16
Primary operable breast cancer treated with PET	6	54
		
**Reason for PET**		
Patient choice	1	17
Unfit for surgery	2	21
Extreme old age	0	4
Dementia	0	5
No reason documented	0	0

). In both age groups, there were patients who opted for PET having been offered a choice of PET or surgery, although the extent of counselling was rarely documented. In 12 out of the 17 older patients who chose PET, significant comorbid conditions were present. Five patients were otherwise fit and well and in only one patient was no reason for PET documented.

### Surgery

#### Complications

There were no deaths related to breast surgery in either age group. There were 16 out of 194 (8.2%) wound-related complications (haematomas and infections) in the 55–69 years age group *vs* nine out of 91 (9.8%) in the older age group. Seroma rates were also similar between the age groups (55–69 years: *n*=37, 19%, 70+: *n*=19, 20.8%). Neither difference was statistically significant. More serious complications were rare in either group. In the older age group there were two systemic complications, one case of heart failure and one of confusion. In the younger age group one patient developed angina.

#### Surgery to the breast: mastectomy *vs* breast-conservation therapy

**Figure 2:**
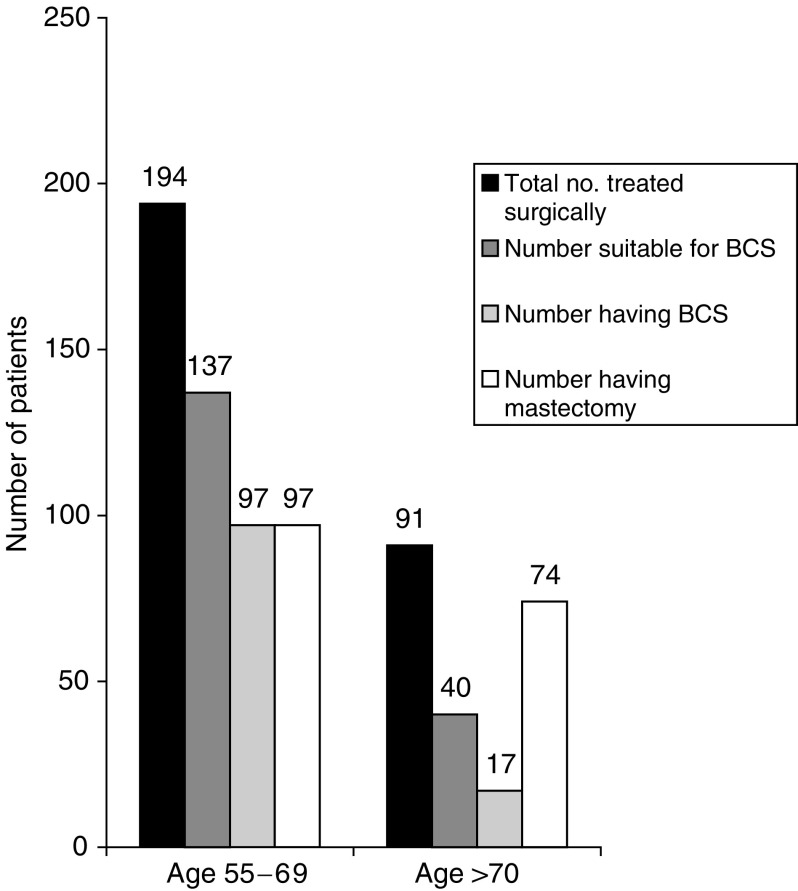
Type of surgery by age group. Graph showing the number of patients in each age group who underwent surgery as their primary treatment. The number of patients suitable for breast-conservation surgery (BCS) was determined by assessing the size of the primary cancer relative to breast size, multifocality, position of tumour relative to the nipple and suitability for adjuvant radiotherapy. The number having mastectomy includes those unsuitable for breast-conservation therapy and those who were suitable but chose mastectomy.

Factors which contraindicate breast-conservation therapy, such as large tumour size relative to breast size, centrally located tumours, multifocality or contraindication to postoperative radiotherapy were identified from the case notes. Using these criteria the number of cases where breast-conservation surgery was an option was compared with the number receiving this treatment option. In the over 70 age group, of the 91 patients who underwent surgery, 40 were suitable for breast conservation. Of these, 23 (57.5%) elected to have a mastectomy as their first surgical procedure. In the 55–69 age group, of the 194 patients who underwent surgery, 137 were suitable for breast conservation. Of these, 40 elected to have mastectomy as their first surgical procedure (20.6%, *P*<0.01, Figure 2

). Therefore, older women were more likely to undergo mastectomy, both for reasons relating to the cancer and also due to patient preference.

#### Surgery to the axilla

UK National Guidelines recommend an axillary staging procedure during surgery for breast cancer (BASO, 1998). Currently this involves axillary clearance or sampling of at least four nodes. Sentinel node biopsy is not currently the standard of care in the UK.

Of the 91 women in the over 70 age group who underwent surgery, two had no axillary surgery because they had DCIS only. Of those with invasive disease undergoing surgery (*n*=89), six (6.7%), had no axillary surgery (*P*<0.01 compared with 55–69 age group), 22 (24.7%) had axillary node sampling and 60 (67.4%) had axillary clearance.

In the younger age group, 194 patients had surgery, 14 had no axillary surgery because they had DCIS only. Of those with invasive disease, one patient had no axillary surgery (0.5%), 47 (26%) had axillary sampling and 131 (72.7%) had axillary clearance.

Of those who had some form of axillary staging 9 (10.7%) had fewer than four nodes assessed in the older age group compared with six (3.37%) of the younger age group (*P*<0.02).

#### Chemotherapy

Reduced chemotherapy tolerance and lack of age-specific evidence of benefit in older women mean that at present adjuvant chemotherapy for patients over the age of 70 years is not standard practice in the UK. However, there are no age-specific exclusion criteria in National Guidelines (BASO, 1998; NICE, 2002). This study found that 69 out of 210 patients in the younger age group received chemotherapy, compared with one out of 167 of the older age group (*P*<0.001).

#### Adjuvant radiotherapy

Radiotherapy to the breast should be utilised as part of breast-conservation therapy (BASO, 1998; NICE, 2002). Radiotherapy to the chest wall should be administered after mastectomy in those cases where the risk of local recurrence is high (BASO, 1998). As in other published reports (Golledge *et al*, 2000; Diab *et al*, 2000; Gajdos *et al*, 2001), older patients were less likely to receive adjuvant radiotherapy following breast-conservation surgery (*P*<0.05). Of those older women eligible for radiotherapy, 12 out of 15 (80%) received it and those that did not were over 85 years of age. Of the younger women, five out of 94 (5.3%) did not receive adjuvant radiotherapy for invasive cancer following WLE. These five patients all had good (*n*=1) or excellent (*n*=4) prognosis tumours and declined radiotherapy after discussion (Table 4).

Chest wall radiotherapy was given to 20 women over 70 and 30 women age 55–69 years (NS, *P*>0.05), although more women who fulfilled the criteria for chest wall radiotherapy (nodal involvement, T3 tumour or a close or positive posterior margin) did not receive it in the older than younger age group (Table 1, *P*<0.01). Radiotherapy doses were the same regardless of age, for both radiotherapy to the breast and chest wall. Radiotherapy response was categorised as none, mild (mild erythema, itching), moderate (moderate erythema, oedema) or severe (severe erythema, moist skin desquamation, pain, significant oedema). There was no significant difference in the incidence or severity of complications with age (data not shown).

#### Adjuvant tamoxifen after surgery

Adjuvant tamoxifen for 5 years after surgery is advised for all women with ER-positive tumours (NICE, 2002). Of 137 eligible younger women, 134 received tamoxifen (97.8%) compared with 63 out of 64 (98.4%) eligible older women (NS, Table 1).

## DISCUSSION

### Tumour stage

This study has demonstrated that stage at presentation of breast cancer is more advanced in older women and that this difference may be largely due to lack of mammographic screening. As has been shown in previous studies, the size of the primary tumour was greater in older women (Diab *et al*, 2000; Golledge *et al*, 2000), and there were trends for worse grade and nodal status. DCIS was more common in younger patients when both symptomatic and screening cases were analysed together, but this difference disappeared when symptomatic cases only were analysed.

Older women were more likely to present with locally advanced and metastatic disease, as has previously been shown (Yancik *et al*, 1989). This excess of advanced disease in the older age group persists even when symptomatic patients only were examined. This may reflect reduced breast awareness among older women, who are less likely to self-examine (Siahpush and Singh, 2002), and may therefore present with more advanced disease (Hill *et al*, 1988).

### Treatment

This study has identified a number of areas where treatment fails to comply with guidelines in older women. Some of these treatment differences were justified, for example, the omission of chemotherapy, which is nonproven as an adjuvant treatment in the elderly (EBCTCG, 1998). However, there is recent evidence that chemotherapy may be of benefit in older women with high-risk cancers which are ER negative (International Breast Cancer Study Group, 2002). A multicentre European study of adjuvant chemotherapy in older patients with poor prognosis breast cancer has been proposed (ACTION, **A**djuvant Cytotoxic **C**hemo**T**herapy **I**n **O**lder Wome**N**. If the present cohort of older women is considered, according to stage and fitness, what percentage would be potential candidates for chemotherapy? The proposed trial may consider patients for adjuvant chemotherapy if they have an NPI>4.4, regardless of ER status. Of the 91 older patients who had surgery, 43 had an NPI of >4.4. Of these patients, five had no comorbidity, 16 had mild comorbidity (e.g. hypertension), four had no documentation relating to comorbidity and the remaining 19 had serious comorbid disease likely to contraindicate chemotherapy (e.g. angina, cardiac failure). Therefore of the total 167 patients, 21 would be potential candidates for adjuvant chemotherapy (12.6%). If we presume that there will be 35 000 new breast cancer diagnoses per year in the UK, of which one-third (11 667) will be over 70. If our findings are generalisable approximately 1500 women across the UK would be potential candidates for chemotherapy annually if such trials find a survival benefit.

Adjuvant radiotherapy was more likely to be omitted in older women. In many of the cases identified, a valid reason was given (e.g. inability to lie flat, previous radiotherapy), although in others, no reason was noted. While there is evidence that radiotherapy may be of less benefit in the elderly because of lower recurrence rates (Benhaim *et al*, 2000), until such studies as the PRIME trial report all suitable patients should be offered radiotherapy, regardless of age.

Primary endocrine therapy is still widely used in older women despite evidence of poor locoregional control. However, in this study, in the majority of cases, its use was justified in that patients were ER positive and had a limited life expectancy or severe comorbidity. There were a small number of cases where fit and healthy women were treated with PET who could have undergone surgery. Many of these women were offered a choice and chose tamoxifen, although it is unclear to what extent this was a truly informed choice.

In summary, this study has demonstrated that women over 70 years of age present with more advanced stage breast cancer and that their treatment more frequently falls outside of agreed National and local guidelines than younger women. This may explain why National mortality statistics show a decline in breast cancer-specific mortality in all other age groups, but are unchanged in the elderly (Quinn *et al*, 2001), where treatment strategies have not altered for the past 20 years. These patients have failed to benefit from the significant advances made in the treatment of breast cancer.

The reasons for treatment of older women outside of guidelines are varied. Some are justified by patient comorbidity and reduced treatment tolerance. In many cases there is insufficient research evidence to guide decision making in this age group and extrapolation from data relating to younger women is the only recourse. This may not always be appropriate because of reduced life expectancy and the slightly different biological behaviour of the disease in older women (Diab *et al*, 2000).
